# Surface Roughness and Hardness of CAD/CAM Ceramic Materials after Polishing with a Multipurpose Polishing Kit: An *In Vitro* Study

**DOI:** 10.1055/s-0042-1758065

**Published:** 2022-12-13

**Authors:** Nareudee Limpuangthip, Ekaluck Poosanthanasarn, Prarom Salimee

**Affiliations:** 1 Department of Prosthodontics, Faculty of Dentistry, Chulalongkorn University, Bangkok, Thailand; 2 Faculty of Dentistry, Chulalongkorn University, Bangkok, Thailand

**Keywords:** CAD/CAM materials, glass ceramic, lithium disilicate, polishing bur, zirconia

## Abstract

**Objective** This article evaluates the effect of multipurpose polishing kit on surface roughness and hardness of three computer-aided design/computer-aided manufacturing (CAD/CAM) ceramic materials at different polishing durations. Weight changes of the polishing bur were also determined.

**Material and Methods** Three CAD/CAM ceramic materials were lithium disilicate glass ceramic (IPS e.max CAD), translucent zirconia (VITA YZ), and zirconia-reinforced lithium disilicate ceramic (Celtra Duo). Ceramics were ground with a diamond bur, and polished with the multipurpose polishing kit (Eve Diacera HP), which comprises coarse and fine polishing burs. Surface roughness value (Ra) was measured using a noncontact optical profilometer ( *n* = 10 per group) after grinding and every 15 seconds of coarse and fine polishing until 60 seconds. The complete polishing Ra was compared with the lab as-received specimens and human enamel. Surface morphology was examined using a scanning electron microscope after 60-second coarse and fine polishing and compared with the lab as-received specimens. Hardness was measured using a Vickers hardness tester on the lab as-received specimens and after the final polishing process ( *n* = 4 per group). Changes in surface roughness and polishing bur weight of each material were analyzed using one-way repeated-measures analysis of variance (ANOVA) and dependent *t* -test. One-way ANOVA was used to detect differences in surface roughness, Vickers hardness, and bur weight among materials within the same polishing duration ( *α* = 0.05).

**Results** From grinding to complete polishing, the greatest Ra reduction was found in VITA YZ, followed by Celtra Duo and IPS e.max CAD. Final Ra values of all ceramics after 60-second fine polishing were not significantly different, and were similar to that of enamel and lab as-received specimens. Vickers hardness of ceramic materials did not change after grinding and polishing. Coarse polishing bur demonstrated the highest weight loss after polishing VITA YZ, followed by Celtra Duo and IPS e.max CAD.

**Conclusion** The multipurpose polishing kit reduced surface roughness of CAD/CAM ceramic materials to the similar level of the lab as-received specimen and enamel regardless of material's hardness. The reductions of surface roughness and a coarse polishing bur weight were highest in VITA YZ, followed by Celtra Duo and IPS e.max CAD.

## Introduction

Due to advancing digital technologies and material evolution, computer-aided design/computer-aided manufacturing (CAD/CAM) ceramic materials have increased in popularity in restorative and prosthodontic dentistry. These materials allow for chairside production and reduce clinical chair-time while maintaining the precision and esthetics of the conventional ceramic materials. 1 Similar to the conventional ceramics, the CAD/CAM ceramic materials can be classified into three types according to the phase present in their chemical composition: glass-matrix ceramic, polycrystalline ceramic and resin-matrix ceramic. 2 Lithium disilicate glass ceramic and stabilized zirconia is the most widely used glass-matrix and polycrystalline ceramic, respectively. To combine the advantages of esthetics and translucency in lithium disilicate glass ceramics and good mechanical properties in zirconia, a zirconia-reinforced lithium silicate (ZLS) has been introduced. ZLS contains zirconium dioxide dissolved in a lithium disilicate-based glass matrix. 3

Clinically, grinding and polishing procedures are important steps for dental ceramic materials, especially for occlusal adjustment. 4 Appropriate polishing prevents crack propagation and subsequent biomechanical failure, reduces biofilm accumulation, 5 prevents excessive wear of opposing and adjacent teeth, 6 7 and enhances esthetics properties such as surface gloss and translucency. 8 9 Currently, a wide variety of commercial dental ceramic polishing kits are available. Porcelain polishing kits, which mainly consist of silica-carbide abrasive, have been used for porcelain and glass ceramic polishing. In contrast, zirconia requires a specific zirconia polishing kit that consists of diamond particles as the main abrasive due to their toughness, and some polishing systems also include a diamond polishing paste for the final polishing step. 6 10 To avoid having to use more than one polishing kit in the clinic, some manufacturers have introduced a multipurpose ceramic polishing kit for polishing all types of ceramic materials, such as Eve Diacera 11 and ZiLMaster. 12 Although EVE Diacera was originally developed for polishing zirconia, 13 the manufacturer subsequently claimed that it could also be used for silica-based ceramic because a high concentration of diamond fillers is incorporated. Thus, using EVE Diacera in polishing silica-based ceramic might be easier due to its lower surface hardness than zirconia-based ceramic materials. However, there is a lack of studies confirming this assumption.

The effect of different polishing systems on the surface properties of the ceramic materials has been investigated. Previous *in vitro* studies determined the effect of different polishing systems on the surface roughness of various ceramic materials by matching between porcelain or zirconia polishing kits and its specified glass ceramic or zirconia material, 8 10 14 and cross-use between zirconia or porcelain polishing kits and glass ceramic, ZLS, or zirconia. 4 15 16 However, the findings varied depending on the polishing systems and ceramic materials. In addition to surface roughness, hardness testing is necessary for ceramic materials. 17 This is because the hardness value reflects the ease of milling and marginal chipping, which are important factors for dental ceramic use. 18 However, there is little information on the surface roughness and hardness of CAD/CAM ceramic materials after polishing with a multipurpose polishing kit at different polishing durations. Also, lack of studies has been conducted to determine the changes of polishing bur weight after the CAD/CAM ceramic materials reach an optimal surface roughness level.

Therefore, the primary objective of this study was to investigate the effect of the multipurpose polishing kit on the surface roughness of three CAD/CAM ceramic materials; lithium disilicate glass-matrix ceramic, zirconia, and ZLS at different polishing durations. The secondary hypotheses were to determine the effects of the multipurpose polishing kit on the hardness of the three materials, and on the changes of polishing bur weight. The primary null hypothesis was that the surface roughness changes of the three CAD/CAM ceramic materials would not be different after polishing with the multipurpose polishing kit. The secondary null hypotheses were that the hardness changes of all the three materials and the polishing bur weight would not be different after final polishing process.

## Materials and Methods

**Fig. 1:**
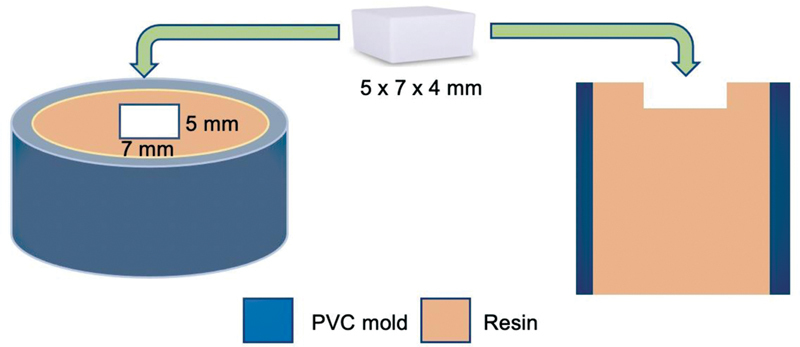
Ceramic specimen embedded in resin.

Table 1 presents the three CAD/CAM ceramic materials used in the present *in vitro* study: lithium disilicate glass ceramic (IPS e.max CAD, Ivoclar Vivadent, Schaan, Lichtenstein), translucent zirconia (VITA YZ, VITA Zahnfabrik H. Rauter GmbH & Co.), and ZLS ceramic (Celtra Duo, Dentsply Sirona), all of which were shade A3. Twelve rectangular (7 × 5 × 4 mm) specimens of each ceramic material were prepared. The IPS e.max CAD and Celtra Duo ceramic blocks were prepared into the predetermined dimensions using a low-speed saw (Isomet 1000; Buehler), and the VITA YZ XT blank was prepared using the CAD/CAM technique with a 20% enlarged dimension of 6.5 × 9 × 5 mm to compensate for sintering shrinkage. The IPS e.max CAD and VITA YZ specimens were sintered in a CEREC SpeedFire furnace (Dentsply Sirona) and a VITA Zyrcomat 6000 MS (VITA Zahnfabrik), respectively, per the manufacturers' recommendation. 19 20 The Celtra Duo specimens were prepared by sintering. 21 A digital vernier caliper (Digimatic; Mitutoyo) was used to measure the specimens' dimensions. The ceramic specimens underwent ultrasonic cleaning (Bransonic model 5210; Branson) in distilled water for 10 minutes and dried with absorbent paper. They were fixed with clear resin in a polyvinyl chloride pipe as illustrated in Fig. 1 .

**Table 1:** Materials and instruments used in this study

Material type	Brand	Manufacturer	Main compositions
CAD/CAM ceramic materials			
Lithium disilicate glass ceramic	IPS e.max CAD (LT) shade A3	Ivoclar Vivadent	SiO _2_ (57–80%), Li _2_ O (11–19%), K _2_ O (0–13%), P _2_ O _5_ (0–11%), ZrO _2_ (0–8%), ZnO (0–8%), Al _2_ O _3_ (0–5%), MgO (0–5%), Coloring oxides (0–8%)
Translucent zirconia	VITA YZ (XT)	VITA Zahnfabrik H. Rauter GmbH & Co. KG	ZrO _2_ (86–91%), Y _2_ O _3_ (8–10%), HfO _2_ (1–3%), Al _2_ O _3_ (0–1%), Pigments (0–1%)
Zirconia-reinforced lithium disilicate	Celtra Duo (LT) shade A3	Dentsply Sirona	SiO _2_ (58%), Li _2_ O (15%), P _2_ O _5_ (5%), ZrO _2_ (10.1%), Al _2_ O _3_ (1.9%), CeO _2_ (2%), Tb _2_ O _3_ (1%)
Polishing instruments			
Fine diamond grinding: 300,000 rpm polishing speed	Fine diamond bur 881F 014 (1.4 × 8 mm dimension)	Hager & Meisinger GmbH	Diamond grit size (27–76 μm)
Multipurpose polishing kit 1. Coarse polishing: 10,000 rpm polishing speed 2. Fine polishing: 10,000 rpm polishing speed	EVE DIACERA HP 1. H2DCmf (4 × 13 mm dimension) 2. H2DC (4 × 13 mm dimension)	EVE Ernst Vetter GmbH	1. Diamond impregnated (25–35 µm) in polyurea 2. Diamond impregnated (3–6 µm) in polyurea

Abbreviations: CAD/CAM, computer-aided design/computer-aided manufacturing; rpm, revolutions per minute.

The sample size was calculated by a software program (G*Power, version 3.1.9.2; Heinrich-Heine-Universität Düsseldorf) using the *F* -test and analysis of variance (ANOVA)-fixed effects, omnibus, one-way. The data from our pilot findings in the three CAD/CAM ceramic materials ( *n* = 3 per group) demonstrated that the surface roughness changes (ΔRa in µm) between grinding and complete fine polishing of IPS e.max CAD, VITA YZ, and Celtra Duo were 0.981, 1.267, and 1.148, respectively, and the average standard deviation (SD) was 0.150. Giving an alpha value of 0.05, a power of 0.80, and the effect size of 0.782, a sample size of 10 for each group was calculated. Two additional specimens were included in each material for scanning electron microscope (SEM) analysis.

**Fig. 2:**
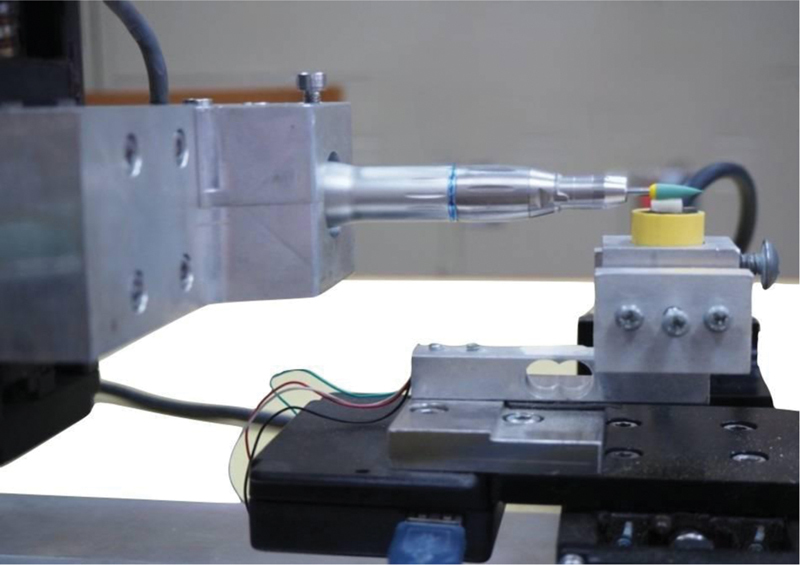
A custom polishing device for controlling the applied force.

The grinding and polishing instruments are described in Table 1 . To ensure standardization of the applied force, a custom pressure control device was used ( Fig. 2 ). The device consists of an electronic control panel, pressure gauge, direction control joystick, load cell, and handpiece connector. The load cell is a transducer that converts the polishing force into a measurable electrical output as a pressure gauge. The direction control joystick controls the polishing bur on the vertical and horizontal axis to provide the desired polishing force. A slow-speed handpiece (NSK Nakanishi Inc.) was used to polish the specimens in a forward-backward direction.

To simulate clinical gross contouring, each ceramic specimen was ground with a fine diamond bur (Meisinger, Hager & Meisinger GmbH) for 15 seconds using a high-speed handpiece (KaVo Dental GmbH) mounted on the custom pressure control device. A 1-N force with a grinding speed of 1 mm/s velocity and 200,000 revolutions per minute (rpm) was applied on each specimen using a gentle stroking forward-backward motion. A new bur was used after grinding five specimens to maintain a consistent amount of diamond grit.

**Fig. 3:**
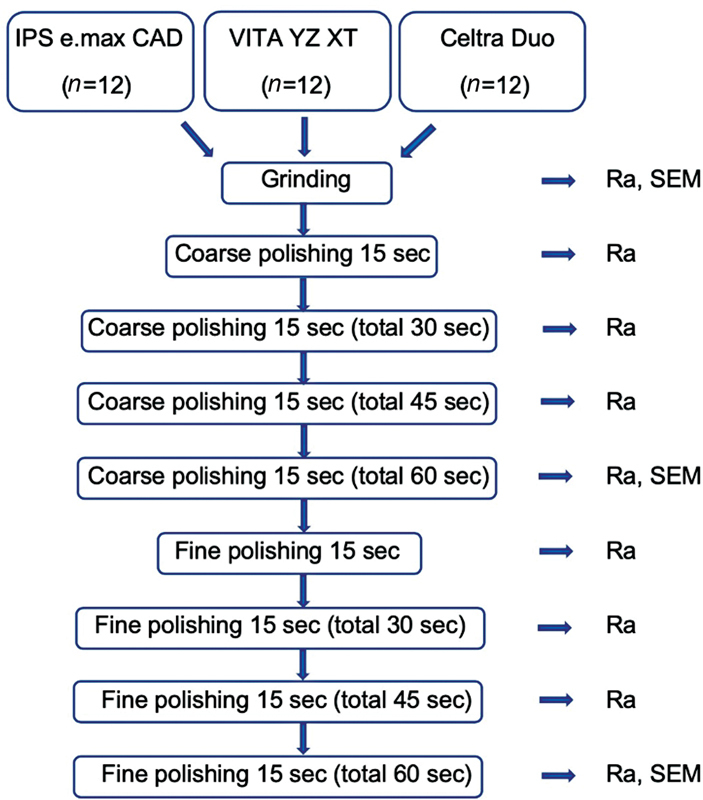
Schematic diagram of the grinding and polishing steps, surface roughness (Ra) measurement, and scanning electron microscope (SEM) analysis.

The polishing process was performed by using a two-step ceramic multipurpose polishing kit (EVE Diacera; EVE Ernst Vetter GmbH) that consisted of a coarse polishing bur (EVE Diacera H2DCmf, green rubber) and a fine polishing bur (EVE Diacera H2DC, pink rubber). The polishing force was controlled at 1 N, 22 and the 10,000 rpm polishing speed was used per the manufacturer's recommendation. 11 The sweeping motion was performed in the same direction as the grinding process for 30 seconds, then rotated 90 degrees and swept perpendicularly to the previous direction for another 30 seconds. The polishing duration consisted of 60 seconds for coarse polishing and 60 seconds for fine polishing, which was obtained from our pilot result that revealed the surface roughness value plateaued after 60 seconds of polishing. A new polishing bur was used after polishing five specimens. Each polishing procedure was paused every 15 seconds for the surface roughness measurement ( Fig. 3 ). The weight of the coarse and fine polishing burs ( *n* = 6) was measured at baseline and after polishing five specimens using a digital analytical balance (Radwag AS220/C/2).

The surface roughness value (Ra) was analyzed using a noncontact optical profilometer (Alicona InfiniteFocusSL) at 50× magnification with a laser to assist in focusing and controlling the same measurement position. Prior to each Ra measurement, the specimen underwent ultrasonic cleaning in distilled water for 5 minutes and air-dried. For each specimen, five areas of 0.4 × 0.4 mm ^2^ were measured, one at the center and the others 1-mm away from the center in four directions. The measurement direction was set perpendicular to the grinding direction, providing a 4-mm evaluation length according to the International Organization for Standardization standard. 23 The mean Ra was calculated from the average value of the five areas. The mean Ra of the three ceramic materials was calculated after grinding and every 15 seconds of coarse and fine polishing until 60 seconds of polishing was achieved.

The complete polishing Ra of the specimens was compared with the lab as-received specimens and human enamel Ra. Five specimens that were prepared in the laboratory were high-gloss polished by a laboratory technician, and their surface roughness was measured. Extracted human teeth were collected and the experimental protocol was approved by the Human Research Ethics Committee of the Faculty (HREC-DCU 2021-067). Thirty extracted human maxillary and mandibular teeth, consisting of 10 incisors, 10 premolars, and 10 molars without visible dental caries or restoration, were collected and stored in 0.1% thymol solution after extraction. The teeth were sectioned mesiodistally and embedded in a resin block exposing the buccal and lingual surfaces for surface roughness measurement, and the mean Ra value was calculated. The specimen surface was examined using a SEM (JSM-6400; JEOL). Three specimens from each ceramic material were randomly selected and were gold-coated in a vacuum prior to the examination.

A Vickers hardness tester (FM-810, Future-Tech Corp) with a load of 100 g for 15 seconds was used to determine the effect of the polishing procedures on the surface hardness of the materials. The hardness was measured at prior to polishing (lab as-received specimens) and after the final polishing process ( *n* = 4 per group). Each sample received five measurements on a polished surface in a linear pattern, and the mean Vickers hardness was calculated.

The data were analyzed using a statistical software program (IBM SPSS Statistics, v28.0; IBM Corp) at *α* = 0.05. The normality of the data was determined using the Shapiro–Wilk test, and parametric statistics were adopted. Two-way repeated-measures ANOVA was used to determine the effect of material types and polishing duration on surface roughness. Because there was an interaction between two independent variables, one-way ANOVA and Tukey post hoc comparison test were used to determine the surface roughness differences between material types after each grinding and polishing duration. One-way repeated-measures ANOVA and Tukey post hoc comparison test were used to determine the surface roughness change in each ceramic material after grinding and polishing. Moreover, one-way ANOVA and Tukey post hoc comparison test were used to compare the surface roughness change (ΔRa), Vickers hardness, and bur weight at baseline, and after polishing of three ceramic materials. Changes in Vickers hardness were determined using the dependent *t* -test, and the bur weight after polishing was compared to the initial value using a one-sample *t* -test.

## Results

After grinding, the Ra values (mean ± SD) in the VITA YZ and Celtra Duo groups were significantly higher those in the of IPS e.max CAD group ( Table 2 ). However, the Ra value in the VITA YZ and Celtra Duo groups were reduced more than the IPS e.max CAD group after 15-second coarse polishing. The surface roughness of all ceramics gradually decreased after coarse and fine polishing. However, the VITA YZ groups' surface roughness was relatively stable from 30-second fine polishing onwards. At all polishing durations, the Ra values of the three ceramic materials were not significantly different. The final Ra value after complete fine polishing were not significantly different from that of enamel (mean Ra ± SD = 0.573 ± 0.167) and the lab as-received specimens.

**Table 2:** Surface roughness (Ra in µm) (mean ± standard deviation) of CAD/CAM ceramic materials and after grinding and polishing and lab as-received specimens ( *n* = 10)

Material types	Lab as-received specimens	Grinding	Coarse polishing				Fine polishing			
			15 s	30 s	45 s	60 s	15 s	30 s	45 s	60 s
IPS e.max CAD	0.698 (±0.084)	1.440 (±0.136) ^aB^	1.121 (±0.201) ^b^	1.095 (±0.164) ^b^	0.969 (±0.122) ^c^	0.936 (±0.131) ^c^	0.864 (±0.141) ^c^	0.756 (±0.065) ^d^	0.701 (±0.089) ^de^	0.647 (±0.110) ^e^
VITA YZ	0.668 (±0.072)	1.838 (±0.205) ^aA^	1.318 (±0.230) ^b^	1.149 (±0.232) ^bc^	1.093 (±0.206) ^c^	0.912 (±0.299) ^d^	0.803 (±0.151) ^d^	0.658 (±0.131) ^e^	0.635 (±0.143) ^e^	0.595 (±0.132) ^e^
Celtra Duo	0.637 (±0.100)	1.663 (±0.113) ^aA^	1.256 (±0.121) ^b^	1.185 (±0.141) ^bc^	1.124 (±0.124) ^c^	1.018 (±0.159) ^d^	0.768 (±0.140) ^e^	0.698 (±0.095) ^f^	0.647 (±0.122) ^fg^	0.593 (±0.088) ^g^

Abbreviation: CAD/CAM, computer-aided design/computer-aided manufacturing.

Note: Different lowercase letters indicate significant difference in rows ( *p* < 0.05). Different uppercase letters indicate significant difference in columns ( *p* < 0.05).

After complete 60-second coarse polishing, the greatest Ra reduction was seen in the VITA YZ group, followed by the Celtra Duo and IPS e.max CAD groups ( Table 3 ). The amount of Ra reduction was not significantly different between the three ceramic materials after continuing from the coarse to complete fine polishing. In the ceramic materials, coarse polishing resulted in a greater Ra reduction compared with fine polishing. After complete fine polishing, the Vickers hardness of the ceramic materials was similar to that of the lab as-received specimens. The Vickers hardness in the VITA YZ group was highest, followed by the Celtra Duo and IPS e.max CAD, respectively.

**Table 3:** Changes in the surface roughness (ΔRa in µm) and Vickers hardness (mean ± standard deviation) of the CAD/CAM ceramic materials after polishing

Ceramic types	Surface roughness reduction (ΔRa, µm)			Vickers hardness	
	Grind-coarse	Coarse-fine	Grind-fine	Lab as-received specimens	After complete polishing
IPS e.max CAD	0.504 (±0.162) ^B^	0.289 (±0.151) ^A^	0.793 (±0.207) ^B^	557.9 (±30.7) ^A^	554.7 (±4.2) ^A^
VITA YZ	0.926 (±0.350) ^A^	0.317 (±0.210) ^A^	1.243 (±0.256) ^A^	1235.9 (±20.3) ^B^	1191.6 (±20.1) ^B^
Celtra Duo	0.645 (±0.173) ^B^	0.425 (±0.143) ^A^	1.070 (±0.128) ^A^	640.7 (±35.6) ^C^	632.4 (±5.1) ^C^

Abbreviation: CAD/CAM, computer-aided design/computer-aided manufacturing.

Note: Different uppercase letters indicate significant difference in columns ( *p* < 0.05).

After polishing five specimens, the weight of the coarse polishing burs was significantly decreased from the baseline value. However, the weight of the fine polishing burs was not ( Table 4 ). The amount of weight loss in the coarse bur was the greatest after polishing VITA YZ, followed by Celtra Duo and IPS e.max CAD.

**Table 4:** The weight of coarse and fine polishing burs (g) (mean ± standard deviation) after 5-specimen polishing ( *n* = 6 per group)

Material types	Bur weight (g)	
	Coarse polishing	Fine polishing
	(Initial weight = 1.445 g)	(Initial weight = 1.432 g)
IPS e.max CAD	1.433 (±0.004) ^A^ a	1.427 (±0.001) ^A^
VITA YZ	1.406 (±0.004) ^B^ a	1.424 (±0.006) ^A^
Celtra Duo	1.429 (±0.013) ^A^ a	1.428 (±0.004) ^A^

Note: Different uppercase letters indicate significant difference in columns ( *p* < 0.05).

a Significant reduction from the initial weight ( *p* < 0.05).

**Fig. 4:**
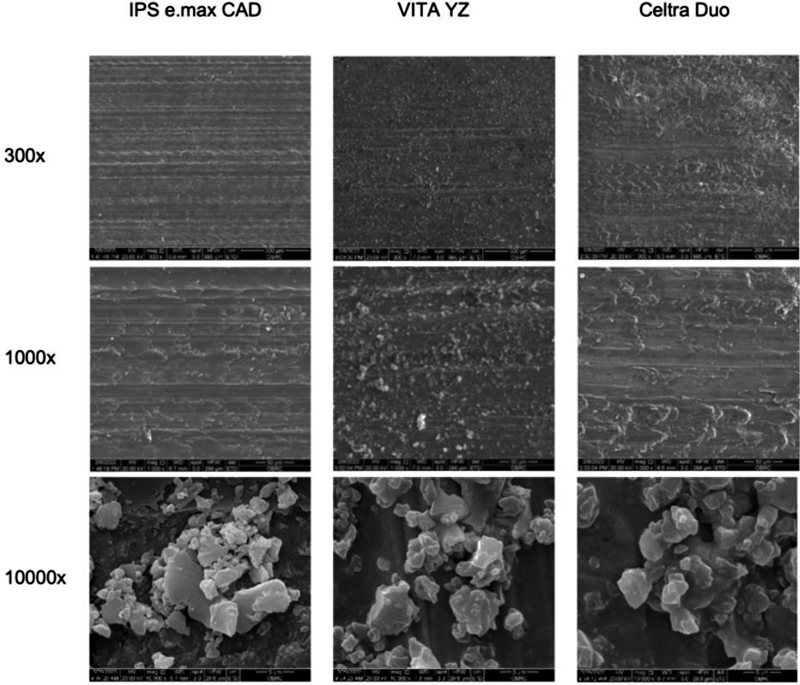
Scanning electron microscope (SEM) of the ground specimens at 300 × , 1,000 × , and 10,000× magnification. IPS e.max CAD and Celtra Duo demonstrated grooves with a scale-like pattern; however, VITA YZ had grooves with a bead-like pattern. At 10,000× magnification, IPS e.max CAD presented a combination of large and small crystal grains with a scale-like glass matrix (upper-left corner), and VITA YZ and Celtra Duo had a more homogenous grain size.

**Fig. 5:**
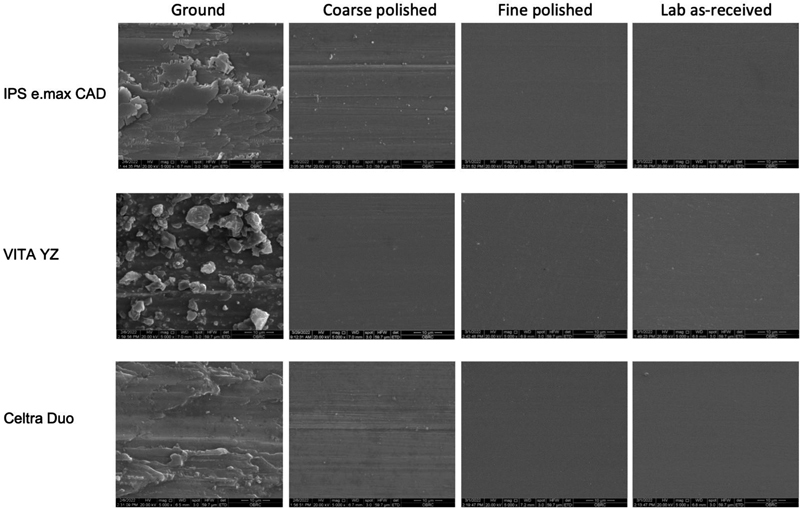
Scanning electron microscope (SEM) of the ground and polished ceramic specimens at 5,000× magnification. Each ceramic demonstrated shallower grooves after coarse polishing and a smoother surface after fine polishing, compared with the lab as-received specimens. The VITA YZ specimen presented a greater roughness reduction after coarse polishing than the other ceramics.

The SEM analyses at 300 × , 1,000 × , and 10,000× magnification demonstrated a wavy-like pattern on the CAD/CAM ceramic materials' surfaces after grinding, with a bead-like wavy pattern on the VITA YZ and a scaly pattern on the Celtra Duo surfaces ( Fig. 4 ). The surface morphology of the materials became progressively smoother from coarse to fine polishing, which was a similar pattern to those of the lab as-received specimens ( Fig. 5 ).

## Discussion

The present study evaluated the effect of the multipurpose polishing kit on the surface roughness of three CAD/CAM ceramic materials; lithium disilicate glass-matrix ceramic, zirconia, and ZLS at different polishing durations. We found that the surface roughness of the three CAD/CAM ceramic materials was significantly reduced after polishing with the multipurpose ceramic polishing kit. The surface roughness at all polishing durations was similar among the materials regardless of material hardness, except after grinding where the surface roughness of VITA YZ specimens was highest, followed by the Celtra Duo and IPS e.max CAD. After complete fine polishing, the greatest reductions in surface roughness and a coarse polishing bur weight were shown in VITA YZ, followed by Celtra Duo and IPS e.max CAD. However, the hardness changes of all materials could not be detected. Based on these results, the null hypothesis was partially rejected.

Types of ceramic material and polishing systems are major factors that affects the material's surface roughness. 4 5 8 15 24 As demonstrated in previous studies, 14 24 the optimal surface roughness of a ceramic material should be at the level of the lab as-received specimen and opposing enamel. Previous *in vitro* studies used the polishing kits that were specific to porcelain/glass ceramic or zirconia, to polish the ceramic materials. In contrast, the present study used EVE Diacera as the multipurpose ceramic polishing system, and the findings revealed that surface roughness of the CAD/CAM ceramic materials after complete polishing was similar to that of enamel and the lab as-received specimens. Smoothening the ceramic surface close to that of enamel decreases the enamel wear of the opposing tooth. 6

Our findings were supported by the study by Matzinger et al which found that the chairside and labside polishing had similar effectiveness in reducing surface roughness of three CAD/CAM materials, comprising IPS e.max CAD, Celtra Duo, and VITA Suprinity which is a ZLS material. 16 A study by Jum'ah et al revealed that polishing the 3Y-translucent zirconia with the two-step EVE Diacera Twist for 90 seconds could reduce surface roughness of the ground material to the similar level of the material undergone glazing. 10 Vichi et al reported that the surface roughness of VITA Suprinity after polishing with Suprinity polishing kits for 60 seconds was significantly lower than that of IPS e.max CAD after polishing with the Optrafine polishing kits. However, the study used specific type of polishing kit for the indicated CAD/CAM ceramic materials. 8 In the present study, the three CAD/CAM ceramic materials had similar surface roughness values after complete polishing; however, their changes across the polishing durations demonstrated different patterns. As demonstrated by Vichi et al, these results may be due to different material's microstructures, 8 and it is expected that the surface roughness would reduce more when the grain size of a polishing bur is larger than that of the ceramic crystalline structure. The crystalline structure of IPS e.max CAD lithium disilicate glass ceramic is needle-like with an 800-nm diameter and 5,000-nm length, while VITA YZ zirconia has an oval-shape with a 815-nm diameter. Celtra Duo, a ZLS material, contains a mixture of shorter needle-like 500 to 700 nm crystals, of which the diameters are smaller than those of VITA YZ. In contrast, the grain size of the coarse and fine polishing bur is approximately 2,500 to 3,500 nm and 300 to 600 nm, respectively. Due to the larger grain size of the coarse polishing bur compared with VITA YZ and Celtra Duo, the two materials demonstrated a greater surface roughness reduction compared with IPS e.max CAD after coarse polishing. The VITA YZ had greater surface roughness reduction than Celtra Duo due to its more homogenous crystal structure. In contrast, IPS e.max CAD presented the least surface roughness reduction among the three materials after complete polishing which could be due to a relatively larger and heterogeneous crystalline structure compared with the polishing bur's grain size. Thus, the efficacy of the multipurpose ceramic polishing kit is rather depended on the polishing bur's grain size in relative with the morphology and size of the ceramic's crystalline structure.

In the present study, the surface roughness was measured after every 15 seconds of polishing, and the total 60-second duration was chosen for coarse and fine polishing because that was when the surface roughness of the ceramic materials reached the plateau level. It was found that the surface roughness of VITA YZ did not reduce beyond 30-second fine polishing, which might be because the surface roughness of VITA YZ was already substantially reduced after coarse polishing. In accordance with our finding, Huh et al found no surface roughness difference between the zirconia undergoing 60- and 120-second polishing durations, 13 which might be because the surface roughness had already reached the plateau level after 60-second of polishing. Vichi et al also reported lower surface roughness value of VITA Suprinity and IPS e.max CAD after 60-second compared with 30-second polishing. Therefore, a polishing duration plays important role in achieving clinically acceptable surface roughness level.

In contrast to surface roughness, the hardness of all the three CAD/CAM ceramics did not change after complete fine polishing. The hardness value of VITA YZ was highest, followed by Celtra Duo and IPS e.max CAD, according to the degree of material toughness. Higher Vickers hardness value reflects milling difficulty, being less prone to marginal chipping, and less permanent deformation of the material surface. 17 18 25 Our findings were consistent with those of Mavriqi et al who found that the Vickers hardness of IPS e.max CAD was lower than that of ZLS ceramic, including Celtra Duo and VITA Suprinity. This is likely because zirconia, which has a finer-grain structure, would be more resistant to permanent deformation compared with ZLS and glass-based ceramic materials. 25 Our findings suggest that the degree of increased surface roughness did not rely on the hardness of the ceramic materials, and the two properties were not correlated. In addition, the multipurpose polishing kit smoothened the ceramic materials without altering the hardness property of the CAD/CAM ceramic materials.

After five-specimen polishing, the coarse polishing bur demonstrated the greatest weight loss after VITA YZ polishing, compared with Celtra Duo and IPS e.max CAD. These results were consistent with the surface roughness reduction in that the more the surface roughness reduction, the greater bur abrasion. In contrast, the amount of weight loss in the fine polishing bur and surface roughness reduction in ceramics were not related. This might be because the diamond abrasives of the fine polishing bur are homogenous and well-embedded in a polyurea core due to their smaller size than the coarse polishing bur. Celtra Duo, a ZLS material, combines the advantage of surface roughness reduction after using the multipurpose ceramic polishing kit as compared to VITA YZ, but similar to IPS e.max CAD in terms of lower polishing bur's abrasion.

Possible confounding variables that could affect surface roughness of ceramic materials were controlled in this study, including the ceramic color and translucency, applied pressure, and polishing device speed. 15 The materials used in this study were A3 color and their translucency was relatively similar. Previous studies found that the translucency of IPS e.max CAD–LT is closed to Celtra Duo–LT, 9 which is closed to VITA YZ–XT. 26 For the polishing procedures in several *in vitro* studies, grinding and polishing was performed by a calibrated operator using finger pressure, 4 8 15 24 with an applied force ranging from 0.4 to 2 N. 8 15 24 However, a custom device is still needed to standardize the applied pressure. 24 Thus, we fabricated a custom polishing machine to standardize the grinding/polishing speed and the applied force exerted on the materials. A 1-N force was chosen as the polishing pressure because it was reported in a previous study, 22 and was also the average value obtained from our pilot result that evaluated the polishing pressure applied by the 20 prosthodontists.

The present study has some limitations. Only one system of the multipurpose ceramic polishing kit and one brand for each type of CAD/CAM ceramic materials were used as a representative, which limited a generalizability of the finding. Thus, our results may be differed from what was achieved clinically because the applied force and polishing duration could be inconsistent among dentists. In addition, surface roughness change can further be affected by the oral environment. Further studies should explore other properties of the CAD/CAM ceramic materials, such as wear of the material and its opposing tooth to comprehensively evaluate the clinical performance of the material.

## Conclusion

After using the multipurpose polishing kit, surface roughness of the CAD/CAM ceramic materials was reduced to the clinically acceptable level compared with the enamel and the lab as-received specimens. Surface roughness and the weight of coarse polishing bur reductions were greatest in VITA YZ, followed by Celtra Duo and IPS e.max CAD. However, the material hardness did not change after final polishing process.
